# *Parabacteroides distasonis* Properties Linked to the Selection of New Biotherapeutics

**DOI:** 10.3390/nu14194176

**Published:** 2022-10-07

**Authors:** Jordan Chamarande, Lisiane Cunat, Nadine Pavlov, Corentine Alauzet, Catherine Cailliez-Grimal

**Affiliations:** 1 SIMPA, Université de Lorraine, F-54000 Nancy, France; 2 Institut Universitaire de Technologie Nancy-Brabois, Université de Lorraine, F-54000 Nancy, France; 3 CHRU de Nancy, Service de Microbiologie, F-54000 Nancy, France

**Keywords:** gut microbiota, *Parabacteroides distasonis*, dysbiosis, immunomodulation, new biotherapeutic product (NBP)

## Abstract

Dysbiotic microbiota is often associated with health issues including inflammatory bowel disease or ulcerative colitis. In order to counterbalance host disorder caused by an alteration in the gut composition, numerous studies have focused on identifying new biotherapeutic products (NBPs). Among the promising NBPs is *Parabacteroides distasonis*, a gut microbiota member part of the core microbiome that recently has received much attention due to the numerous beneficial properties it brings to its host. In this study, the properties linked to the selection of NBPs were screened in 14 unrelated *P. distasonis* strains, including resistance to gastric conditions, adherence (Caco-2 model), transepithelial resistance (Caco-2 model), and immunomodulation, on nontreated and LPS-stimulated cells (HT-29 and peripheral blood mononuclear cells (PBMCs)). This approach allowed for the identification of five strains that combined almost all the in vitro biotherapeutic properties tested. However, all the *P. distasonis* strains induced the overproduction of proinflammatory cytokines on PBMCs, which was counteracted by the overproduction of the anti-inflammatory cytokines. Among these five strains, two particularly retained our attention as a potential NBP, by showing strong health-promoting function, the lowest overproduction of proinflammatory cytokines on PBMCs, and no detrimental effect on the host.

## 1. Introduction

The human digestive tract gathers approximately 100 trillion bacteria that are part of the gut microbiota (GM; [1]). The genetic information encompassed in this ecosystem is 150 times more important than that of the human genome with around 10 million nonredundant genes described [2]. The important roles played by both humans and bacteria in this symbiotic relation have led to the rising popularity of concepts such as superorganism or holobiont, i.e., the sum of a metazoan host organism and its associated microbes functioning as an inextricably intertwined single unit [3,4].

The progression of sequencing methods allowed for the characterization and understanding of the contribution of the GM to the host’s well-being. Many lines of evidence show that the cooperation between the GM and its host is essential to regulate the development and function of the immune, metabolic, and nervous systems. In addition, the intestinal microbiota also creates a protective barrier against external pathogens and participates in maintaining the structure and integrity of the gastrointestinal tract [3,5,6]. On the other hand, the immune system has to control and preserve its relationships with the GM. In the long run, the GM can modulate host behavior and the nervous system’s functions through dynamic and bidirectional communication along the gut–brain axis [7].

Many factors can influence gut composition, including diet, environmental exposure, antimicrobial therapies, or also physical/psychological stress [8]. Alterations in the gut microbiota composition, the so-called dysbiosis, often lead to chronic disorders, including Crohn’s disease and ulcerative colitis, which are the most prevalent forms of inflammatory bowel diseases (IBDs; [9]). Other pathologies such as autism spectrum disorders, type I diabetes, or even a change in social behavior are correlated with an alteration in the GM [3,8].

To overcome the problems related to microbiota imbalance, many studies focused on the discovery and characterization of new biotherapeutic products (NBPs; [10]). In addition to the traditional probiotic organism, defined as a “live organism that when administrated in adequate amount confer a health benefit on the host” [11,12], and prebiotic organism, defined as “a substrate that is selectively utilized by host microorganisms conferring a health benefit” [13], new therapeutic approaches such as fecal microbial transplantation (FMT) have emerged as putative microbiota-targeting therapies [14,15]. Another approach consists of the identification and selection of novel and disease-specific health-promoting bacteria [16,17,18,19].

*Parabacteroides distasonis,* a Gram-negative strictly anaerobic bacterium belonging to the *Tannerellaceae* family in the *Bacteroidetes* phylum, has recently received considerable attention for showing anti-inflammatory properties and activities in decreasing weight gain, hyperglycemia, and hepatic steatosis in ob/ob and high-fat-diet-fed mice [17,18,19,20,21]. This species has also been shown to display anticancer properties and attenuates tumorigenesis mediated by a reduction in TLR4, MYD88, and Akt signaling and the stimulation of apoptosis [22,23]. In addition, *P. distasonis* harbors a small but specific repertoire of degrading enzymes, making it a specialist fibrolytic bacterium [24,25].

In this study, we investigated the properties linked to the selection of NBPs of 14 unrelated strains of *P. distasonis*. For this purpose, we characterized the adhesion, resistance to gastric conditions, epithelial barrier strengthening, and immunomodulation capacities of *P. distasonis* strains. Immunomodulation was investigated in both the epithelial barrier and immune system cell models.

## 2. Materials and Method

### 2.1. Bacterial Strains and Culture Conditions

A total of 14 *P. distasonis* strains, including 13 nonredundant clinical and clonally unrelated (as described in [26]) isolates collected by the clinical microbiology laboratory of the University Hospital of Nancy, France, and the type strain *P. distasonis* DSM 20701^T^ were tested.

*Lactobacillus rhamnosus* GG ATCC 53103 (LGG) was used as a positive control for the adhesion capacity assays [27].

*Escherichia coli* ATCC 25922 was used as a positive control for the transepithelial electrical resistance [28].

The strains were stored at −80 °C in an appropriate broth supplemented with 15% glycerol. Prior to assay, *P. distasonis* strains were streaked onto Brucella agar plates supplemented with 5% of defibrinated sheep blood (BBA; Oxoid, Thermo Fisher Diagnostics, Dardilly, France), 1% of hemin (Sigma-Aldrich, Saint Quentin Fallavier, France), and 1% of vitamin K1 (Sigma-Aldrich, Saint Quentin Fallavier, France). After incubation at 37 °C for 48 h in anaerobic conditions, the strains were subcultured in a Schaedler broth for 24 h in anaerobic conditions. LGG and *E. coli* were streaked onto De Man, Rogosa, and Sharpe (MRS) or Luria–Bertani (LB) agar, respectively, and incubated at 37 °C for 24 h in aerobic conditions. Further subcultures were performed in a Schaedler broth for 24 h in aerobic conditions.

### 2.2. Cell Lines and Culture Conditions

The human intestinal cell lines Caco-2 and HT-29 were obtained from Pr. Isabelle Chevalot (UMR 7274, Reactions and Chemical Engineering Laboratory, CNRS, Lorraine University, Nancy, France) and Dr. Henri-Pierre Lassalle (Lorraine Institute of Oncology, Nancy, France), respectively. Caco-2 cells were grown in a Dulbecco’s modified Eagle medium—high glucose (HG-DMEM with 4500 mg/L glucose and L-Glutamine; Sigma-Aldrich, Saint Quentin Fallavier, France) supplemented with 10% heat-inactivated (56 °C, 30 min) fetal bovine serum (FBS; Gibco, Evry, France), 100 U/mL penicillin–0.1 mg/mL streptomycin (Sigma-Aldrich, France), and 10 µM 4-(2-hydroxyethyl)-1-piperazineethanesulfonic acid (HEPES; Sigma-Aldrich, Saint Quentin Fallavier, France). HT-29 cells were grown in a McCoy 5A medium (Fisher Scientific, Illkirch, France) supplemented with 10% heat-inactivated FBS, 10 µM HEPES buffer, and 100 U/mL penicillin–0.1 mg/mL streptomycin.

The peripheral blood mononuclear cells (PBMCs) were extracted from the blood samples of five healthy donors following the density gradient separation method using a lymphocyte separation medium (Eurobio scientific, Les Ulis, France) and a Leucosep tube (Greiner bio-one, Les Ulis, France). The obtained PBMCs were then washed and resuspended in an RPMI-1640 medium (Gibco, Evry, France) supplemented with L-glutamine (2 mM; Sigma-Alrich, Saint Quentin Fallavier, France), pyruvate (1 mM; Gibco, Evry, France), and 10% heat-inactivated FBS.

### 2.3. Tolerance to Gastric Conditions

The survival kinetics of the strains in simulated gastric juice (SGJ) were measured during 2 h of incubation, as previously described by [29]. The SGF was prepared with KCL 6.9 mM, HCl 15.6 mM, KH_2_PO_4_ 0.9 mM, NaHCO_3_ 25 mM, NaCl 47.2 mM, MgCl_2_ 0.1 mM, (NH_4_)_2_CO_3_ 0.5 mM (all from Sigma-Aldrich, Saint Quentin Fallavier, France) and sterilized through filtration. The pH was then adjusted to 3 using HCl 1 M, and CaCl_2_ and porcine pepsin (Sigma-Aldrich, Saint Quentin Fallavier, France) were added to achieve a final concentration of 0.075 mM and 2.000 U/mL, respectively, in the final digestion mixture. The bacterial suspension was centrifugated (2000 rpm, 10 min), washed twice with PBS, and standardized at 10^9^ CFU/mL. Then, 950 µL of SGF (with pepsin and at pH 3) was inoculated with 50 µL of the bacterial suspension to reach a final concentration of 5 × 10^7^ CFU/mL and incubated at 37 °C for 2 h with sampling at 0 and 120 min. Bacterial viability was measured by the numeration of a serial dilution plated on BBA plates after 48 h incubation in anaerobiosis. The death rate was calculated by dividing the number of CFU/mL at 120 min by the CFU/mL measured at time zero, as the 14 studied strains have been previously shown to survive under aerobic conditions without any indication of mortality for 6 h [26].

### 2.4. Bacterial Adhesion to Caco-2 Cell Line

For epithelial adherence assay, 12-well tissue culture microplates were seeded with 6 × 10^4^ Caco-2 cells per well for 21 days in order to have a homogeneous and polarized cell monolayer. After 21 days, the cells were washed twice with PBS and inoculated with *P. distasonis* suspension (HG-DMEM without antibiotic) at 10^9^ CFU/mL. The plates were then incubated for 3 h at 37 °C under a 5% CO_2_ atmosphere and washed 4 times with PBS to remove nonadherent bacteria. Caco-2 cells and the adhered bacteria were resuspended in 1 mL of 0.1% Triton 100X (Sigma-Aldrich, Saint Quentin Fallavier, France) with a cell scraper. The DNA was then extracted, and a specific region of the 16S rRNA of *P. distasonis* was amplified and quantified using qPCR as previously described [26].

### 2.5. Transepithelial Electrical Resistance

The intestinal epithelial barrier permeability was evaluated by measuring the transepithelial electrical resistance (TEER) with a Millicell ERS-2 system (Millipore, Burlington, USA). Briefly, Caco-2 cells were seeded in a 24-well Transwell^®^ insert filter (polycarbonate membrane with 0.4 µm pore size; Sarstedt, Germany) at a density of 10^5^ cells/cm² in HG-DMEM supplemented as previously described in cell culture conditions. The medium was renewed every two days during the first week and every day during the second week, followed by a measurement of the TEER until day 14 when constant and optimal transepithelial resistance was reached (TEER > 300 Ω/cm²). *P. distasonis* strains were prepared with HG-DMEM and added to the apical side of the membrane at a bacteria-to-cell ratio of 10:1. The TEER was measured just after adding the bacteria (T0) and after 24 h of incubation at 37 °C, 5% CO_2_ (T24). The TEER variation was measured by comparing the electrical resistance obtained after 24 h of incubation with the initial resistance.

### 2.6. Immunomodulation Assay on HT-29 and PBMC Cells

Briefly, 24-well microplates were inoculated with 5 × 10^4^ HT-29 cells/mL. The plates were then incubated at 37 °C under a 5% CO_2_ atmosphere until reaching confluence. HT-29 cells were then washed two times with PBS and stimulated with *E. coli* LPS (Sigma Aldrich, Saint Quentin Fallavier, France) and *P. distasonis* at a final concentration of 10^7^ CFU/mL. To define *E. coli* LPS concentration to use, assays ranging from 1 ng/mL to 100 ng/mL were performed. The final concentration selected was 1 ng/mL, which induced the same response as other concentrations (data not shown). After a 4 h incubation with LPS and bacteria, the supernatants were collected and stored at −20 °C until cytokine (IL-8) measurement, performed with an IL-8 human ELISA kit (Thermofisher Scientific, Illkirch, France).

For PBMC stimulation, 24-well microplates were inoculated with 1 × 10^6^ of the extracted cells/mL. PBMCs were stimulated with 100 ng/mL of *E. coli* LPS (Sigma-Aldrich, Saint Quentin Fallavier, France) and *P. distasonis* at a final concentration of 10^7^ CFU/mL. After 18 h incubation with LPS and bacteria, the supernatants were collected and stored at −20 °C until cytokines’ (IFN-γ, IL-1β, IL-1, IL-12p70, TNF-α, IL-1RA, and IL-10) measurement with a Milliplex Luminex^®^ 200™ System (Merck Millipore, Molsheim, France) using the Luminex™ Xmap (Multi-Analyte Profiling) technology.

### 2.7. Statistical Analysis

A one-way analysis of variance (ANOVA) was used to group the samples according to their adhesion capacities and to determine the strains that significantly impact Caco-2 TEER or HT-29/PBMC cytokine production. The *p* values < 0.05 were considered statistically significant. All statistical analyses were carried out with the XLSTATs program version 2022.2.1 (Addinsoft, Paris, France).

## 3. Results

### 3.1. Survival Capacity of P. Distasonis to Gastric Conditions

**Figure 1:**
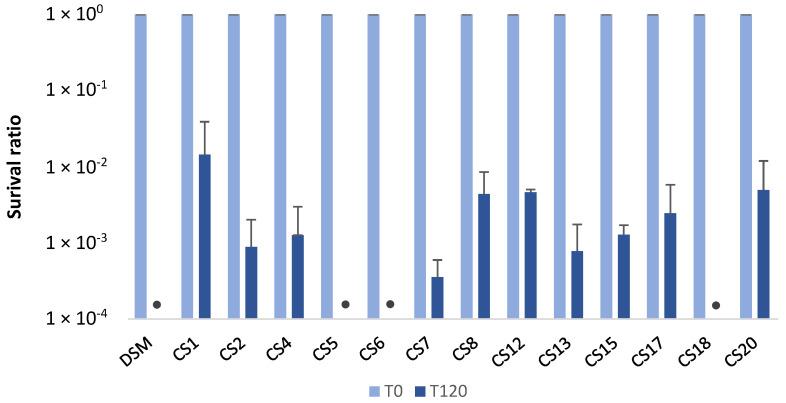
Bacterial survival of 14 *P. distasonis* strains to gastric stress after 120 min of incubation. The survival is shown as the ratio of the CFU/mL at T120 to the CFU/mL at T0. DSM: *P. distasonis* DSM 20701^T^; CS: clinical strains; • indicates the samples in which the CFU/mL reached the detection threshold (<10). All the data represent mean ± standard error from at least two independent experiments performed with each strain in triplicate (n = 2 × 3).

None of the 14 *P. distasonis* strains tested totally resisted after 2 h in gastric conditions (Figure 1). Among them, a two-log decrease in viability was observed for CS1 and between two- and four-log for CS2, 4, 7, 8, 12, 13, 15, 17, and 20. Other strains, namely DSM and CS5, 6, and 18, seemed more sensitive to gastric conditions, as no colony was counted after 2 h in SGJ.

### 3.2. Adhesion Capacities of P. Distasonis Strains to Caco-2 Cells

**Figure 2:**
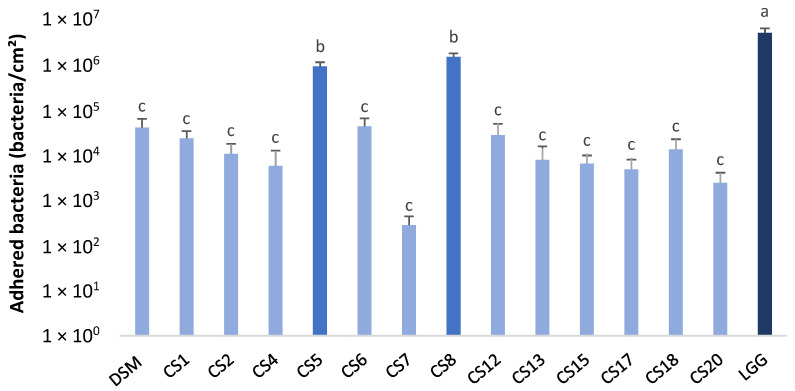
Adhesion capacity of 14 strains of *P. distasonis* and LGG (positive control) on Caco-2 cells after 3 h of incubation at 37 °C under a 5% CO_2_ atmosphere. DSM: *P. distasonis* DSM 20701^T^; CS: clinical strains. Colors coupled with a, b and c indicate the significantly different groups after ANOVA, *p* < 0.05. All the data represent mean ± standard error or observations from triplicate (n = 3).

The results obtained for the adhesion of *P. distasonis* strains on Caco-2 cells revealed three groups that were significantly different (Figure 2). The first one was denoted as (a) with the positive control LGG, the second one was denoted as (b) with CS5 and CS8, and the third one (c) included 12 of the 14 *P. distasonis* strains. All the tested strains showed the capacity to adhere to biotic support after 3 h of incubation. The adhesion capacity of the 10^9^ inoculated bacteria/mL ranged from 2.84 × 10^2^ adherent bacteria/cm² (CS7) to 1.51 × 10^6^ adherent bacteria/cm² (with CS8 showing an adhesion capacity relatively close to the one of the positive control LGG).

### 3.3. Effect of P. Distasonis on Caco-2 Monolayer Integrity

**Figure 3:**
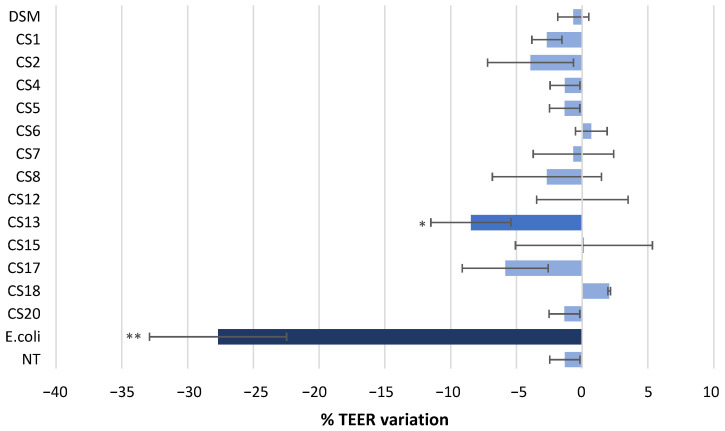
Effect of *P. distasonis* strains on Caco-2 monolayer TEER. *E. coli* ATCC 25922 was used as positive control. The results are shown as % of TEER variation after 24 h of incubation. DSM: *P. distasonis* DSM 20701^T^; CS: clinical strains. Statistical analysis was performed using an ANOVA test in comparison with % variation in nontreated (NT) Caco-2 (* *p* < 0.05, ** *p* < 0.01). All the data represent mean ± standard error from triplicate (n = 3).

Among the 14 *P. distasonis* tested, only CS13 significantly modified the TEER values of the Caco-2 monolayer after 24 h of incubation by inducing a diminution of the TEER of approximately 8% (Figure 3). CS18 was the only strain that tended to increase the monolayer integrity with a TEER augmentation of 2%. The 12 other strains neither significantly reinforced nor damaged the Caco-2 monolayer junctions.

### 3.4. Effect of P. Distasonis on IL-8 Production by Untreated and LPS-Stimulated HT-29 Cells

**Figure 4:**
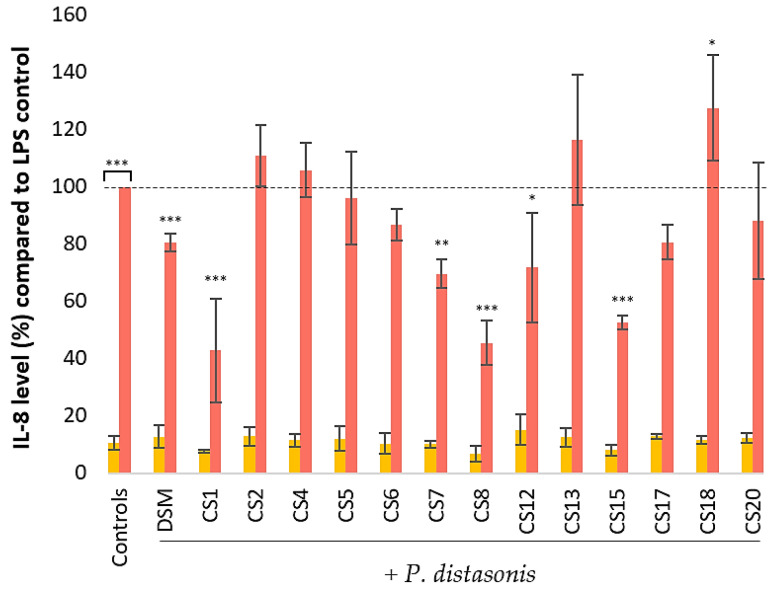
Effect of 14 strains of *P. distasonis* on IL-8 production by nonstimulated and LPS-stimulated (4 h; 1 ng/mL) HT-29 cells. The IL-8 levels were measured using ELISA. DSM: *P. distasonis* DSM 20701^T^; CS: clinical strains. Controls: untreated and *E. coli* LPS-stimulated HT-29; yellow: no LPS stimulation; red: HT-29 stimulated with *E. coli* LPS. Dotted line represents 100% equal to HT-29 responses to *E. coli* LPS stimulation. Statistical analyses were performed using an ANOVA test (* *p* < 0.05, ** *p* < 0.01, *** *p* < 0.001). The nontreated HT-29 control and all co-stimulation with *E. coli* LPS and *P. distasonis* assays were compared with the LPS-stimulated HT-29 control, which represents 100% of IL-8 released. The single stimulations of HT-29 with *P. distasonis* were compared with the nontreated HT-29 control. All the data represent mean ± standard error from triplicate (n = 3).

The LPS-stimulated HT-29 cells were distinguished from the nonstimulated HT-29 cells by their overexpression of IL-8 (Figure 4). Among the 14 *P. distasonis* tested, none of them induced a response similar to that of LPS from *E. coli*. The co-stimulation of HT-29 cells with *E. coli* LPS and *P. distasonis* strains induced several responses that seemed to be strain-dependent. DSM, CS1, 7, 8, 12, and 15 significantly reduced the inflammation generated by LPS. By contrast, CS2, 4, 5, 6, 13, 17, and 20 did not modify the IL-8 produced in response to *E. coli* LPS. CS18 was the only strain that significantly increased the inflammatory response of LPS.

### 3.5. Immunomodulation of P. Distasonis on Untreated and LPS-Stimulated PBMC

**Figure 5:**
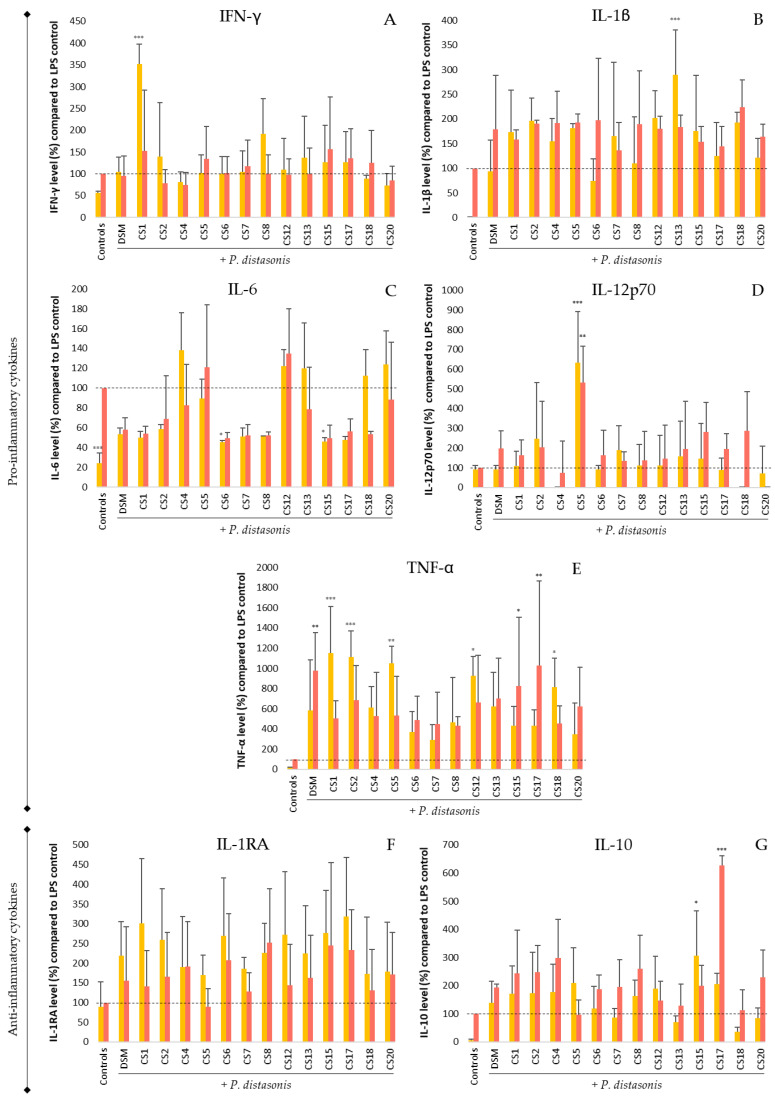
Immunomodulation properties of *P. distasonis* on nonstimulated and LPS-stimulated (18 h; 100 ng/mL) PBMCs. The cytokine levels were measured using Luminex technology. (**A**): IFN-γ; (**B**): IL-1β; (**C**): IL-6; (**D**): IL-12p70; (**E**): TNF-α; (**F**): IL-1RA; (**G**): IL-10. DSM: *P. distasonis* DSM 20701^T^; CS: clinical strains. Controls: nontreated and *E. coli* LPS-stimulated PBMC; yellow: no LPS stimulation; red: *E. coli* LPS stimulation. Dotted line represents 100% equal to PBMC responses to *E. coli* LPS stimulation. Statistical analyses were performed using ANOVA in comparison with the LPS-stimulated PBMC assay that represents 100% of cytokines released (* *p* < 0.05, ** *p* < 0.01, *** *p* < 0.001). All the data represent mean ± standard error from at least three samples (n ≥ 3).

The immunomodulation of *P. distasonis* on PBMCs was investigated via the production of pro- and anti-inflammatory cytokines, namely IFN-γ, IL-1β, IL-6, IL-12p70, and TNF-α (pro-), as well as IL-1RA and IL-10 (anti-) (Figure 5).

Concerning the proinflammatory cytokines, *E. coli* LPS stimulation caused the overproduction of each of them except IL-12p70.

IFN-γ production by PBMCs (Figure 5A) also increased in the presence of all the tested *P. distasonis* strains with notably 250% overproduction when stimulated with CS1 compared with *E. coli* LPS. However, LPS caused only a slight increase (about 50%) in the IFN-γ production, compared with the untreated cells. No significant difference was observed for stimulation with both LPS and *P. distasonis*, compared with individual stimulations.

For IL-1β production (Figure 5B), all the *P. distasonis* strains provoked the overproduction of the cytokine similar to that observed for LPS with CS13, which induced a level of overproduction significantly more important than *E. coli* LPS. The co-stimulation tended to display IL-1β overproduction compared with untreated PBMCs, as shown for individual stimulations with the LPS control and *P. distasonis*.

All but two *P. distasonis* strains increased IL-6 production (Figure 5C) similarly to the LPS control stimulation. Strains CS6 and CS15 also increased the production of IL-6 by PBMCs but significantly less than *E. coli* LPS. The results obtained for the co-stimulation correspond to the *P. distasonis* single stimulation with a strain-dependent increase in IL-6 production.

IL-12p70 (Figure 5D) production was only modified by CS5, which significantly increased the production by approximately 600% in both nonstimulated and LPS-stimulated PBMCs.

Finally, TNF-α production (Figure 5E) increased by *P. distasonis*, compared with the LPS control, which only caused a slight increase in TNF-α production, compared with nontreated PBMCs. Indeed, although strain-dependent, all tested strains provoked a PBMC response at least 300% superior to that of *E. coli* LPS including five responses significantly higher. The co-stimulation was strain-dependent, but three strains (DSM, CS15 and CS17) seemed to potentialize TNF-α production with an important increase in production compared with the single stimulation.

As regards the anti-inflammatory cytokines, PBMC IL-1RA production (Figure 5F) was not influenced by *E. coli* LPS, while IL-10 production (Figure 5G) increased.

Furthermore, 5 of the 14 *P. distasonis* strains (DSM, CS1, CS6, CS15, and CS17) engendered the overproduction of IL-1RA, up to 200%, compared with the LPS control, but not significantly. The co-stimulation did not modify IL-1RA production in comparison with individual stimulations.

As observed for the LPS stimulation, all *P. distasonis* strains tended to increase the IL-10 production of PBMCs. Among them, only CS15 engendered IL-10 production significantly more important than the LPS control. The co-stimulation seemed to amplify the response but not significantly, compared with the LPS control. However, one response was surprisingly different with an augmentation of the IL-10 production by 600%, compared with *E. coli* LPS (CS17).

## 4. Discussion

The predominant role of GM in the health and disease of its host is now well-defined. A disequilibrium in its composition can lead to the development of many chronic diseases. Thus, the gut microbiota has become a prime target in treatments in which the first objective is to return to a more balanced microbial community. For this purpose, many studies focus on performing deeper research into both classical probiotics *Lactobacillus* and *Bifidobacterium* and identifying new candidates for NBPs [17,18,30,31,32]. Numerous studies identified *P. distasonis* as a promising candidate, showing anti-inflammatory and anticancer properties and displaying beneficial activities for its host [17,18,19,20,21,22,23]. In all these studies, only a few strains of *P. distasonis* have been investigated including strains isolated from mice GM, and anti-inflammatory properties are often associated with the investigation of the production of only a few cytokines. However, some of these results and previous work performed in our laboratory have shown the significant inter-strain variability of *P. distasonis* in terms of response.

In the present study, we investigated the properties of 14 clinical strains of *P. distasonis* linked to the selection of new biotherapeutics. The selected properties were the resistance of *P. distasonis* to gastric conditions, its in vitro adherence capacity to the intestinal epithelial cell model, its effect on the epithelial monolayer integrity, and its immunomodulation on both the epithelial and immune system cell models with and without stimulation with *E. coli* LPS. The investigation of such characteristics allows for the discovery of a range of abilities from the intake of bacteria to its potential benefits for the host.

In vitro models that simulate digestion processes are widely used to study the gastrointestinal behavior of pharmaceuticals to ensure the good (bio)delivery of the studied element despite gastric conditions, i.e., low pH and peptidases. Among the 14 *P. distasonis* strain tested, none of them completely resisted the gastric conditions, and 10 out of the 14 strains showed gastric resistance properties. These results coincide with those of previous studies that highlighted the resistance of two *P. distasonis* strains among the five tested, also displaying an inter-strain variability [18,19]. However, the acidic conditions of the stomach can be neutralized by the addition of sodium bicarbonate to the bacterial pellets, allowing for the potential use of nonresistant *P. distasonis* strains as NBPs.

After passing through the rough conditions of the upper gastrointestinal tract and reaching the small and large intestine, bacteria have to colonize it. The adherence capacities of *P. distasonis* on Caco-2 cells were investigated. All the tested strains had the capacity to adhere to the epithelial cells. However, an inter-strain variability was observed with notably two strains showing an adhesion capacity as important as the positive control LGG. These differences could be explained by the presence of diverse external proteinaceous structures at the surface of each strain, the expression of which was potentially controlled by the inversions of the promoter region, leading to phase variable synthesis. Among these structures are pili-like and fimbriae-like structures from the Mfa and Fim systems, respectively, as well as capsular polysaccharides [33].

The epithelial layer of the gastrointestinal tract forms a physical and biochemical barrier against all external bacteria, including pathogens. The integrity of this barrier is critical for the well-being of the host, and its alteration is often linked with IBDs [34]. The effect of *P. distasonis* on the Caco-2 cell integrity was investigated. As expected from a commensal bacterium, 13 of the 14 *P. distasonis* strains did not impact the monolayer integrity compared with the positive control *E. coli* ATCC 25922, as previously described for three strains [17]. The last strain, CS13, significantly altered the Caco-2 monolayer integrity (8% compared with nontreated Caco-2), although less than the positive control (by 28%). Contrarywise, CS18 slightly reinforced the epithelial monolayer by approximately 2%. This result could be explained by the stimulation of the expression of protein-encoding genes Occludin and ZO-1, which are part of a tight junction multiprotein complex, as previously described for two *P. distasonis* strains [19]. This complex is composed of at least 40 different transmembrane and cytoplasmic proteins with expressions that can be modulated by active factors released by the gut microbiota members [28,34].

*P. distasonis* has also been shown to display anti-inflammatory capacities in both in vitro and in vivo models. In our in vitro experiments, none of the tested strains induced the overproduction of IL-8 in HT-29 cells, and 6 of the 14 strains statistically reduced the LPS-induced IL-8 inflammation, as previously observed [17,22]. These results put forward the potential anti-inflammatory activity of *P. distasonis* on the intestinal epithelium barrier model.

In PBMCs, the production levels of the proinflammatory cytokines IFN-γ, IL-1β, IL-6 and TNF-α were increased by almost all the *P. distasonis* strains as well as *E. coli* LPS. Moreover, the *P. distasonis* treatment during *E. coli* LPS-induced inflammation did not reduce the proinflammatory cytokine production of PBMCs. These results, contrary to HT-29 results, suggest a proinflammatory response of the immune system cells due to *P. distasonis* stimulation, although with strain variability. These results do not agree with the findings of previous works performed in mice models, where *P. distasonis* was shown to counteract acute TNBS-induced colitis by modulating the expressions of IL-1β, IL-6, and TNF-α, and a decrease in the expression of IL-6 in DSS-induced chronic colitis was also highlighted [19,21]. However, these results, as well as the results shown in this work, exposed an important inter-strain variability that could explain these differences in terms of immunomodulation.

*P. distasonis* also provoked the overproduction of anti-inflammatory cytokines IL-1RA and IL-10. The lack of modification in IL-12p70 production by almost all the *P. distasonis* strains and the overproduction of IL-10 induced by *P. distasonis* coincide with previous works [18,19]. IL-1RA is a specific IL-1 receptor antagonist that competitively binds to the same receptor as IL-1β without inducing a cellular signal, thereby blocking IL-1β-mediated cellular changes [35]. Consequently, the IL-1β production induced by *P. distasonis* could be possibly countered by the production of IL-1RA. In the same way, IL-10 is a cytokine that represses the expression of inflammatory cytokines such as IL-1, IL-6, and TNF-α by activating macrophages and downregulating the proinflammatory cytokine receptors [36].

The integration of the data generated here and in a previous study [26] highlighted five strains (CS1, CS7, CS8, CS12 and CS15) that combined 10 of the 12 in vitro biotherapeutic properties tested (Table 1). CS1, although possessing the strongest adhesion capacity and resistance to gastric conditions, caused significant overproduction of proinflammatory cytokines IFN-γ and TNF-α by PBMCs. In the same way, the CS12 significantly increased TNF-α production. CS15 was the only strain, along with CS6, to increase IL-6 production significantly less than *E. coli* LPS, but this strain and CS17 tended to strongly increase TNF-α production, especially with *E. coli* LPS. CS7 and CS8 for their part only caused slight overproduction of IL-6 and were among the strains that never induced the overproduction of proinflammatory cytokines compared with *E. coli* LPS. Moreover, these strains, and mainly CS8, showed strong adhesion and biofilm formation capacities and were among the strains that significantly reduced IL-8 production by LPS-stimulated HT-29 cells.

## 5. Conclusions

Numerous studies provide evidence of the beneficial roles of *P. distasonis* within GM. However, only few strains are studied, although important inter-strain variability has been observed. In this study, we investigated the properties linked to the selection of the NBPs of a wide diversity of *P. distasonis* strains. Although almost all the tested strains had the ability to adhere and not damage the Caco-2 monolayer, an important inter-strain variability was observed for SGJ tolerance and immunomodulation on nontreated and LPS-stimulated HT-29 cells and PBMCs. None of the tested strains induced the overproduction of proinflammatory cytokines on the epithelial cell model, and 6 of the 14 strains significantly reduced the inflammation induced by *E. coli* LPS. However, all the strains induced the overproduction of proinflammatory cytokines in the immune system cell model, which could be counteracted by the increased production of anti-inflammatory cytokines, although further study is required. Such data confirmed the promising use of *P. distasonis* as a biotherapeutic product. However, further investigations have to be performed in order to better understand its strain variability and the absence of pernicious side effects.

## Figures and Tables

**Table 1:** Summary of the beneficial and nondetrimental properties of the 14 strains of *P. distasonis* investigated in this study. Color code: beneficial/nondetrimental (green) or detrimental (red) properties.

		*P. distasonis*															
		DSM 20701^T^	CS1	CS2	CS4	CS5	CS6	CS7	CS8	CS12	CS13	CS15	CS17	CS18	CS20		
**Abiotic**	Homo-aggregation																
	Adhesion																Strongest adhesion capacity
	Biofilm formation																Strongest biofilm formation capacity
	SGJ tolerance																Strongest SGJ tolerance
**Caco-2**	Adhesion																Strongest adhesion capacity
	maintain TEER																Enhance or damage TEER
**HT-29**	IL-8 production (NT)																
	Decrease IL-8 production (LPS)																Strongest anti-inflammation properties
**PBMC**	Pro-inf. production (NT)																Never increase more than LPS control
	Anti-inf. production (NT)																Increase IL-10 significantly more than LPS control
	Decrease pro-inf. production (LPS)																
	Increase anti-inf. production (LPS)																Increase IL-10 significantly more than LPS control
NT: non-treated; LPS: LPS-stimulated cells			Positive effect or capacity								Negative effect or capacity						

## Data Availability

Data are contained within the article.
